# Subcellular differential expression of Ep-ICD in oral dysplasia and cancer is associated with disease progression and prognosis

**DOI:** 10.1186/s12885-016-2507-7

**Published:** 2016-07-16

**Authors:** Raj Thani Somasundaram, Jatinder Kaur, Iona Leong, Christina MacMillan, Ian J. Witterick, Paul G. Walfish, Ranju Ralhan

**Affiliations:** Alex and Simona Shnaider Laboratory, Laboratory Medicine in Molecular Onocolgy, Mount Sinia Hospital, Room 6-318, 600 University Avenue, Toronto, ON M5G 1X5 Canada; Department of Otolaryngology, Head and Neck Surgery, Mount Sinai Hospital, Joseph and Wolf Lebovic Health Complex, 600 University Avenue, 6-500, Toronto, ON M5G 1X5 Canada; Department of Pathology and Laboratory Medicine, Mount Sinai Hospital, Toronto, ON M5G 1X5 Canada; Department of Laboratory Medicine and Pathobiology, University of Toronto, Toronto, ON M5S 1A8 Canada; Joseph and Mildred Sonshine Family Centre for Head and Neck Diseases, Mount Sinai Hospital, Toronto, ON M5G 1X5 Canada; Department of Otolaryngology – Head and Neck Surgery, Alex and Simona Shnaider Laboratory in Molecular Oncology, Mount Sinai Hospital, Joseph & Wolf Lebovic Health Complex, 600 University Avenue, 6-500, Toronto, ON M5G 1X5 Canada; Department of Otolaryngology – Head and Neck Surgery, University of Toronto, Toronto, ON M5G 2N2 Canada; Department of Medicine, Endocrine Division, Mount Sinai Hospital and University of Toronto, Joseph & Wolf Lebovic Health Complex, Room 413-7, 600 University Avenue, Toronto, ON M5G 1X5 Canada

**Keywords:** Ep-ICD, EpCAM, Oral lesion, Dysplasia, Squamous cell carcinoma, Oral cancer, Prognosis

## Abstract

**Background:**

Identification of patients with oral dysplasia at high risk of cancer development and oral squamous cell carcinoma (OSCC) at increased risk of disease recurrence will enable rigorous personalized treatment. Regulated intramembranous proteolysis of Epithelial cell adhesion molecule (EpCAM) resulting in release of its intracellular domain Ep-ICD into cytoplasm and nucleus triggers oncogenic signaling. We analyzed the expression of Ep-ICD in oral dysplasia and cancer and determined its clinical significance in disease progression and prognosis.

**Methods:**

In a retrospective study, immunohistochemical analysis of nuclear and cytoplasmic Ep-ICD and EpEx (extracellular domain of EpCAM), was carried out in 115 OSCC, 97 oral dysplasia and 105 normal oral tissues, correlated with clinicopathological parameters and disease outcome over 60 months for oral dysplasia and OSCC patients. Disease-free survival (DFS) was determined by Kaplan-Meier method and multivariate Cox regression analysis.

**Results:**

In comparison with normal oral tissues, significant increase in nuclear Ep-ICD and membrane EpEx was observed in dysplasia, and OSCC (*p* = 0.013 and < 0.001 respectively). Oral dysplasia patients with increased overall Ep-ICD developed cancer in short time period (mean = 47 months; *p* = 0.044). OSCC patients with increased nuclear Ep-ICD and membrane EpEx had significantly reduced mean DFS of 33.7 months (*p* = 0.018).

**Conclusions:**

Our study provided clinical evidence for Ep-ICD as a predictor of cancer development in patients with oral dysplasia and recurrence in OSCC patients, suggesting its potential utility in enhanced management of those patients detected to have increased risk of progression to cancer and recurrence in OSCC patients.

## Background

Head and neck cancer is the sixth most prevalent cancers accounting for approximately 600,000 new cases annually worldwide [1]. Oral squamous cell carcinoma (OSCC) is the major subtype of head and neck cancer and accounts for two-thirds of the cases occurring in least developed countries [2]. OSCCs are often preceded by development of clinically distinct oral lesions; on an average about one percent of oral lesions transform into cancer annually [3, 4]. Histologic assessment of a biopsy with evidence of dysplasia is used for determining the risk of malignant transformation; increasing grade of dysplasia (mild/moderate/severe) has been associated with a high rate of malignant transformation. However, dysplasia grading is subjective, not often associated with malignant transformation; some dysplastic lesions may remain static or even regress, while the non-dysplastic lesions may occasionally become malignant. Accurate assessment of oral dysplasia and identification of lesions at high risk of malignant transformation remains a major clinical challenge and is of immense importance for identifying patients in whom early intervention will lead to more effective disease management. The key to early detection and effective management of the disease lies in better understanding of the molecular mechanisms implicated in malignant transformation of oral lesions with dysplasia. Furthermore, despite improvements in treatment strategies the prognosis of OSCC patients remains largely unsatisfactory, due to loco-regional recurrence. The 5-year survival rates are about 50 %, and the prognosis of advanced cases has not improved much over the past 4 decades [2]. At present, the most important prognostic factors include histological tumor grade, stage, depth of tumor invasion and involvement of regional lymph nodes at the time of diagnosis.

Epithelial cell adhesion molecule (EpCAM) is a transmembrane glycoprotein expressed in several human epithelial tissues and frequently overexpressed in cancer, progenitor, and stem cells [5]. EpCAM consists of an extracellular epidermal growth factor-like (EGF) domain (EpEx), thyroglobulin domain, transmembrane region, and a short intracellular domain (Ep-ICD) [6, 7]. In normal cells, EpCAM appears to be sequestered in tight junctions and is therefore less accessible to antibodies, whereas in cancer cells it is widely distributed on the cell surface and has therefore been explored as a surface-binding site for therapeutic antibodies [8–11]. EpCAM is involved in cell signaling, migration, proliferation, cell cycle regulation, and cancer metastasis. and has been widely investigated for its diagnostic and therapeutic potential as it is expressed in the majority of human epithelial cancers, including breast, colon, esophageal, gastric, hepatic, head and neck, prostate, pancreas, ovarian and lung cancer [12–23]. Increased EpCAM expression has been found to be a poor prognostic marker in breast and gall bladder carcinomas [24, 25]. In contrast EpCAM expression in colorectal and gastric cancer is associated with favorable prognosis [26, 27]. This paradoxical association of EpCAM expression with prognosis in different cancers is supported by functional studies of EpCAM biology using in vitro and in vivo cancer models as well. Taken together these studies suggest that the impact of EpCAM expression in human cancers is likely to be context dependent [28]. EpCAM expression based assay has been FDA approved and widely used to detect circulating tumor cells in breast cancer [29]. Due to its high-expression and association with poor prognosis, EpCAM has been widely explored as a potential target for antibody-based immunotherapies [30]. EpCAM expression has been used to predict response to anti-EpCAM antibodies in breast cancer patients [30–32]. Surprisingly clinical trials of anti-EpCAM antibodies targeting the EpEx domain have shown limited efficacy [31, 33]. These paradoxical outcomes are potentially explainable by the regulated intramembranous proteolysis of EpCAM, resulting in oncogenic signaling by its intracellular domain, Ep-ICD [34]. Previously, we reported accumulation of Ep-ICD is frequently detected in ten epithelial cancers, including breast and prostate [35, 36]. In thyroid carcinomas nuclear Ep-ICD (Ep-ICD_Nuc_) accumulation predicted poor prognosis and was elevated in patients with anaplastic tumors [36]. Recently, a dynamic expression of EpCAM was reported in esophageal cancer throughout tumor progression [16].

We hypothesized that alterations in Ep-ICD and EpEx sub-cellular localization in membrane, cytoplasm and nucleus could influence oral cancer pathogenesis and may correlate with clinical outcome in these patients. In this study, we determined the clinical significance of alterations in expression and sub-cellular localization of Ep-ICD and EpEx protein in oral tumorigenesis.

## Methods

### Study design

This retrospective study of Ep-ICD and EpEx using OSCC and dysplasia patients’ tissue blocks stored in the archives of Department of Pathology and Laboratory Medicine and their anonymized clinical data was approved by the Mount Sinai Hospital (MSH) Research Ethics Board, Toronto, Canada, prior to commencement. The study was conducted according to the Reporting Recommendations for Tumor Marker prognostic studies (REMARK) guidelines and a retrospectively written research, pathological evaluation, and statistical plan [37]. The patients granted informed written consent for their tissue samples to be archived and used for research purposes and publication of research findings.

### Patients

Patient demographic, clinical, and pathological data were recorded in a pre-designed Performa as described previously [38].

#### Inclusion criteria

Patients with histopathological evidence of dysplasia or squamous cell carcinoma of the oral cavity and a known clinical outcome were inducted into the study.

#### Exclusion criteria

Patients diagnosed with dysplasia or squamous cell carcinoma of the oral cavity but with no available follow-up data or patients diagnosed with dysplasia concomitant with OSCC at the first visit were excluded from the study.

### Specimen characteristics

The patients’ charts with clinico-pathological diagnosis of OSCC from 2000 to 2008 were retrospectively reviewed to obtain the clinical information and follow-up data in the Department of Pathology, MSH. Information regarding gender, age, site of lesions at the time of the initial diagnosis of dysplasia or OSCC was documented in the clinical database. Following the above inclusion and exclusion criteria, archived tissue specimens of OSCC patients (*n* = 115, median age: 61 years; range: 30–92 years) undergoing curative cancer surgery during the period 2000–2008 were inducted into this study and 105 normal tissues and 97 oral dysplasia were also obtained from the archived tissue bank at MSH, Canada. All OSCC patients were treated as per the National Comprehensive Cancer Network (NCCN) guide lines for head and neck cancers [38].

### Survival data

Malignant transformation versus non-transformation of oral dysplastic lesions was considered to be the clinical outcome of the patients with oral dysplasia. Follow-up period was defined as the interval from the time when patient underwent first biopsy to the non-transformation at last consultation (for censored observations) or to cancer development (for uncensored observations). Dysplasia patients were monitored for a maximum period of 60 months (mean 36.4 months and median 38 months). Dysplasia to cancer development was observed in 22 of 97 (23 %) patients.

After completion of primary treatment OSCC patients were followed up for up to 60 months (mean 32.8 months and median 29.5 months). Notably, recurrence was observed in 28 % patients. Disease-free survivors were defined as patients free from clinical and radiological evidence of local, regional, or distant relapse at the time of the last follow-up. In the current study, recurrence of the cancer versus no recurrence of OSCC was considered to be the clinical outcome of the patients. Follow-up period was defined as the interval from the time when patient underwent first surgery to recurrence of cancer (for uncensored observations) or no recurrence at last consultation (for censored observations).

### Immunohistochemistry (IHC)

The histopathologic diagnosis of all cases were re-examined by the oral pathologists at MSH. Tissue microarrays (TMAs) were constructed using 100 of 115 OSCCs, 99 of 105 normal oral tissues and 95 of 97 oral dysplasias as reported [39], while the remaining tissues were used as individual sections for immunostaining. Formalin-fixed paraffin embedded sections (4 μm thickness) were used for Ep-ICD and EpEx immunostaining as described [28]. In brief, for EpEx following deparaffinization and rehydration, antigen retrieval was carried out using a microwave oven in 0.01 M citrate buffer, pH 3.0 and endogenous peroxidase activity was blocked by incubating the tissue sections in hydrogen peroxide (0.3 %, v/v) for 20 min. For Ep-ICD, the tissue sections were de-paraffinized by baking at 62 °C for 1 h in vertical orientation, treated with xylene and graded alcohol series, and the non-specific binding was blocked with normal horse or goat serum. Rabbit anti-human Ep-ICD monoclonal antibody from Epitomics Inc. (Burlingame, CA) was used. The α-Ep-ICD antibody 1144 has been used in our previous study of Ep-ICD expression in thyroid carcinoma and other epithelial cancers [36]. Anti-EpCAM monoclonal antibody EpEx (MOC-31, AbD Serotec, Oxford, UK) recognizes an extracellular component (EGF1 domain- aa 27–59) in the amino-terminal region [40]. The sections were incubated with either α-Ep-ICD rabbit monoclonal antibody 1144 (dilution 1:1500) or mouse monoclonal antibody MOC-31 (dilution 1:200) for 60 min, followed by biotinylated secondary antibody (goat anti-rabbit or goat anti-mouse) for 20 min. The sections were finally incubated with VECTASTAIN Elite ABC Reagent (Vector Laboratories, Burlington, ON, Canada) and diaminobenzidine was used as the chromogen. Tissue sections were then counterstained with hematoxylin. Negative controls comprised of oral tissue sections incubated with isotype specific IgG in place of the primary antibody, and positive controls (colon cancer tissue sections known to express Ep-ICD) were included with each batch of staining for both Ep-ICD and EpEx.

### Evaluation of immunohistochemical staining

Each TMA slide or individual tissue section was evaluated for Ep-ICD and EpEx immunoreactivity using a semi-quantitative scoring system for both staining intensity and the percentage of positive epithelial cells as described [39]. Immunopositive staining was evaluated in randomly selected five areas of the tissue section. For Ep-ICD and EpEx protein expression, sections were scored as positive if epithelial cells showed immunostaining in the nucleus/cytoplasm when observed independently by three of us, who were blinded to the clinical outcome (slides were coded and the scorers did not have prior knowledge of local tumor burden, lymphonodular spread, and grading of tissue samples). The tissue sections were scored based on the % of immunostained cells as: 0–10 % = 0; > 10–30 % = 1; > 31–50 % = 2; > 51–70 % = 3 and > 71–100 % = 4. Sections were also scored semi-quantitatively on the basis of staining intensity as negative = 0; mild = 1; moderate = 2; intense = 3. Finally, a total score was obtained by adding the score of percentage positivity and intensity therefore giving a score range from 0 to 7 [39]. We also we calculated the final scores based on the multiplication of the two factors: score of percentage positivity and the intensity of each of the tissue section, and performed the statistical analysis. Each tissue section was scored for cytoplasmic Ep-ICD (Ep-ICD_Cyt_) and Ep-ICD_Nuc_ as well as for membrane EpEx (EpEX_Mem_) following both these scoring methods.

### Statistical analyses

The immunohistochemical data were subjected to statistical analysis with SPSS 22.0 software (SPSS, Chicago, IL) as described previously [41]. A two-tailed p-value was used in all analyses and a p-value < 0.05 was considered statistically significant. Chi-square analysis was used to determine the relationship between Ep-ICD and EpEx expression and the clinicopathological parameters. Disease-free survival was analyzed by the Kaplan-Meier method and multivariate Cox regression. Hazard ratios (HR), 95 % confidence intervals (95 % CI), and p-values were estimated using the log-rank test. Disease-free survival or clinical recurrence was considered to be the endpoint of the study. The cut-offs for statistical analysis were based upon the optimal sensitivity and specificity obtained from the Receiver operating curves as described [35]. For the IHC total score obtained by adding the score of percentage positivity and intensity for Ep-ICD_Nuc_, an IHC score cut-off value of ≥ 2 was defined as immunopositive for all tissues analyzed for statistical analysis. Ep-ICD_Cyt_ positivity was considered positive with an IHC cut-off value of ≥ 4. EpEx_Mem_ positivity was defined as EpEx_Mem_ IHC score of ≥ 2. A cut-off value of ≥ 3 was used for the combination of Ep-ICD_Nuc_ and EpEx_Mem_ positivity. For oral dysplasia, the overall Ep-ICD positivity was defined as the sum of Ep-ICD_Nuc_ + Ep-ICD_Cyt_ with a cut-off value of ≥ 6. For the IHC scores based on the multiplication of the score of percentage positivity and the intensity of each of the tissue section, the cut-offs for positivity were defined as - Ep-ICD_Nuc_ ≥1, Ep-ICD_Cyto_ ≥ 3 and EpEx_Mem_ ≥ 1.

## Results

The clinicopathological parameters of 115 OSCCs and 97 dysplasia patients are summarized in Table 1. The median age of patients with OSCCs was 61 years (range 30 – 92) and dysplasia was 60 years (range 30 – 88). AJCC pTNM stages III and IV comprised of a large proportion of tumors in the study cohort.

**Table 1:** Clinicopathological characteristics of OSCC patients

Study subjects (*N* = 317)	*N*
Normal	105
Dysplasia	97
OSCC	115
Dysplasia (*N* = 97)	*N* (%)
Age years (range, median)	30–88, 60
Gender	
Male	51 (53 %)
Female	46 (47 %)
Follow-up outcome	
Positive	22 (23 %)
Negative	61 (63 %)
Data not available	14 (14 %)
OSCC (*n* = 115)	
Age years (range, median)	30–92, 61
Sex	
Male	73 (63 %)
Female	42 (37 %)
AJCC pTNM classification	
I	21 (18 %)
II	19 (17 %)
III	23 (20 %)
IV	35 (30 %)
Unknown	17 (15 %)
Extra capsular invasion	
Positive	18 (16 %)
Negative	97 (84 %)
Perineural involvement	
Positive	33 (29 %)
Negative	82 (71 %)
Vascular involvement	
Positive	16 (14 %)
Negative	99 (86 %)
Follow-up outcome	
Positive	32 (28 %)
Negative	61 (53 %)
Data not available	22 (19 %)

### Immunohistochemical analysis of Ep-ICD and EpEx expression in oral tissues

**Fig. 1:**
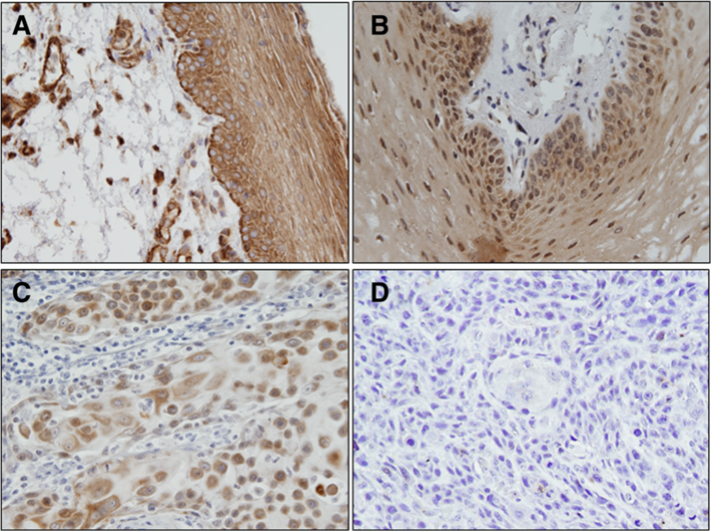
Immunohistochemical analysis of Ep-ICD in oral tissues. Paraffin-embedded sections of histologically normal mucosa, oral dysplasia and OSCC were stained using anti-Ep-ICD monoclonal antibody as described in Methods section. Panel presents representative photomicrographs of Ep-ICD staining. **a** Shows predominantly Ep-ICD_Cyt_ staining in normal oral mucosa with some stromal staining; **b** Increased cytoplasmic and nuclear staining is observed in dysplasia; **c** OSCC also shows cytoplasmic and nuclear staining; **d** No immunostaining was observed in tissue sections used as negative controls where the primary antibody was replaced by isotype specific IgG; while a known OSCC showing Ep-ICD_Nuc_ and Ep-ICD_Cyt_ was used as a positive control (Data not shown); (**a**, **b**, **c**, **d**, original magnification x 200)

**Fig. 2:**
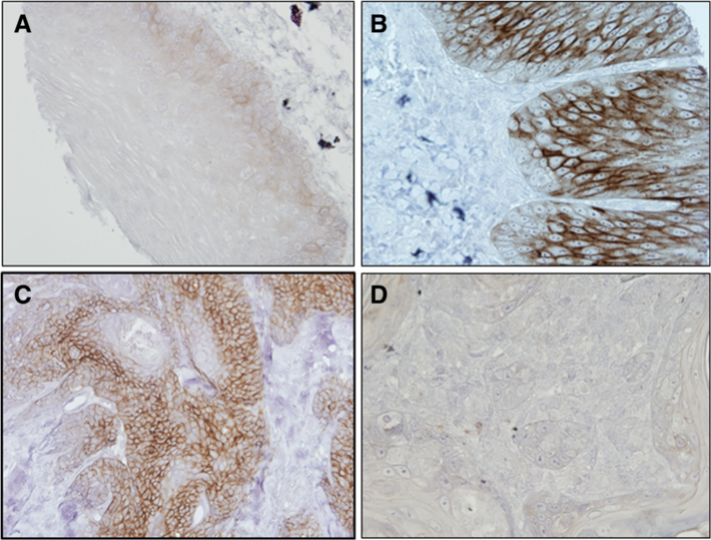
Immunohistochemical analysis of EpEx in oral tissues. Paraffin-embedded sections of histologically normal mucosa, oral dysplasia and OSCC were stained using anti-EpEx monoclonal antibody as described in Methods section. Panel represents (**a**) normal oral mucosa showing no detectable EpEx_Mem_ immunostaining; **b** Oral dysplasia showing intense EpEx_Mem_ staining; **c** OSCC section illustrating reduced EpEx_Mem_ in tumor cells; **d** OSCC section used as a negative control, showing no EpEx immunostaining in tumor cells where the primary antibody was replaced by isotype specific IgG (A-D original magnification x 200)

To determine the clinical significance of Ep-ICD and EpEx in development of oral cancer, its expression was analyzed in OSCC, oral dysplasia and histologically normal tissues and the findings are summarized in Table 2. Representative photomicrographs of Ep-ICD and EpEx immunostaining in normal oral tissue, oral dysplasia and OSCC are presented in Figs. 1 and 2 respectively. Figure 1a shows predominantly Ep-ICD_Cyt_ staining in normal oral mucosa with some of the stromal components also showing immunostaining, increased cytoplasmic and nuclear staining is observed in dysplasia (Fig. 1b) and OSCC also shows cytoplasmic and nuclear staining (Fig. 1c), while a known OSCC showing Ep-ICD_Nuc_ and Ep-ICD_Cyt_ was used as a negative control (Fig. 1d), where the primary antibody was replaced by isotype specific IgG and no immunostaining was observed. No detectable EpEx_Mem_ immunopositivity was observed in normal oral mucosa (Fig. 2a), increased EpEx_Mem_ immunostaining was observed in dysplasia (Fig. 2b), and reduced EpEx_Mem_ staining was observed in OSCC (Fig. 2c), while no detectable EpEx_Mem_ immunostaining was observed in OSCC tissue section used as negative control where the primary antibody was replaced by isotype specific IgG (Fig. 2d).

**Table 2:** Analysis of Ep-ICD and EpEx expression in Normal oral mucosa, Dysplasia and OSCC

				Comparison with normal tissues			Comparison with dysplastic tissues		
	*N*	*n*	%	*p*-value	O.R	95 % C.I.	*p*-value	O.R	95 % C.I.
Ep-ICD Nuclear									
Normal	105	38	36.19						
Dysplasia	97	52	53.61	0.013	2.037	1.16–3.58			
OSCC	115	98	85.22	<0.001	10.164	5.30–19.49	<0.001	4.99	2.60–9.57
Ep-ICD Cyto									
Normal	105	87	82.86						
Dysplasia	97	81	83.51	0.90	1.05	0.50–2.19			
OSCC	115	92	80	0.59	0.83	0.42–1.64	0.512	0.79	0.39–1.60
EpEX membrane									
Normal	105	15	14.29						
Dysplasia	97	37	38.14	<0.001	3.70	1.87–7.33			
OSCC	115	28	24.35	0.06	1.93	0.97–3.86	0.03	0.52	0.29–0.94

Significant increase in Ep-ICD_Nuc_ (*p* = 0.013) and EpEx_Mem_ (*p* < 0.001) was observed in dysplasia as compared to normal oral tissues (Table 2). OSCC patients also showed significant increase in Ep-ICD_Nuc_ (*p* < 0.001) as compared to normal oral tissues (Table 2). The loss of EpEx_Mem_ has been correlated with epithelial- mesenchymal transition and increased aggressive phenotype as well as cancer progression. Hence we compared the expression of EpEx and Ep-ICD between dysplasia and OSCC. Notably, significant loss of EpEx_Mem_ was observed in OSCC as compared to dysplasia (*p* = 0.03) (Table 2). The final IHC scores based on the multiplication of the score of percentage positivity and intensity of each of the tissue section also gave similar results (Table 3).

**Table 3:** Analysis of Ep-ICD_(%positivity*intensity)_ and EpEx_(%positivity*intensity)_ expression in Normal oral mucosa, Dysplasia and OSCC

				Comparison with normal tissues			Comparison with dysplastic tissues		
	*N*	*n*	%	*p*-value	O.R	95 % C.I.	*p*-value	O.R	95 % C.I.
Ep-ICD Nuclear									
Normal	105	38	36.19						
Dysplasia	97	52	53.61	0.013	2.037	1.16–3.58			
OSCC	115	98	85.22	<0.001	10.164	5.30–19.49	<0.001	4.99	2.60–9.57
Ep-ICD Cyto									
Normal	105	87	82.86						
Dysplasia	97	81	83.51	0.90	1.05	0.50–2.19			
OSCC	115	92	80	0.59	0.83	0.42–1.64	0.512	0.79	0.39–1.60
EpEX membrane									
Normal	105	15	14.29						
Dysplasia	97	37	38.14	<0.001	3.70	1.87–7.33			
OSCC	115	28	24.35	0.06	1.93	0.97–3.86	0.03	0.52	0.29–0.94

Cut-offs: Ep-ICDNuc – 1, Ep-ICD Cyto – 3 and EpEX Membrane – 1

### Prognostic analysis of Ep-ICD and EpEx in oral dysplasia and OSCC patients

**Fig. 3:**
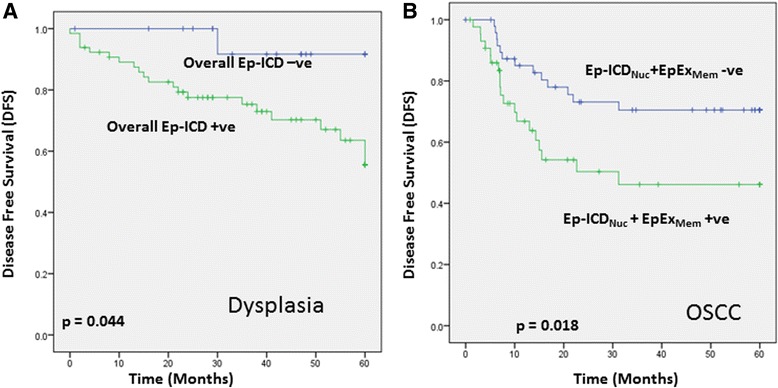
Kaplan Meier survival analysis of Ep-ICD in Oral Dysplasia and OSCC patients. **a** Dysplasia patients with increased overall (nuclear and cytoplasmic) Ep-ICD score had significantly reduced mean cancer free survival of 47 months as compared to patients with low overall Ep-ICD score (mean cancer free survival = 57.5 months; *p* = 0.044); **b** OSCC patients with increased Ep-ICD_Nuc_ and EpEx_Mem_ score had significantly reduced mean disease free survival (DFS) of 33.7 months as compared to patients with decreased Ep-ICD_Nuc_ and EpEx_Mem_ score (mean DFS = 46.3 months; *p* = 0.018)

The relationships between the alterations in expression of Ep-ICD_Nuc_, overall Ep-ICD (combination of Ep-ICD_Nuc_ and Ep-ICD_Cyt_), EpEx_Mem_ and a combination of Ep-ICD_Nuc_ and EpEx_Mem_ with clinical outcome of oral dysplasia and OSCC patients were determined by Kaplan Meier survival analysis over a follow up period of 60 months to investigate their utility as prognostic markers for dysplasia and OSCC. Dysplasia patients with increased overall Ep-ICD had significantly shorter mean cancer free survival of 47 months as compared to patients with low overall Ep-ICD (mean DFS = 57.5 months; *p* = 0.044, Fig. 3a). OSCC patients with increased combination of Ep-ICD_Nuc_ and EpEx_Mem_ had significantly reduced mean DFS of 33.7 months as compared to patients with low Ep-ICD_Nuc_ and EpEx_Mem_ score (mean DFS = 46.3 months; *p* = 0.018, Fig. 3b). Among the OSCC cases, Cox multivariate regression analysis showed combination of Ep-ICD_Nuc_ and EpEx_Mem_ and Extra capsular invasion to be the most important prognostic markers for reduced DFS (*p* = 0.003, HR = 4.01, C.I. = 1.64–9.83 and *p* = 0.004, HR = 4.14, C.I. = 1.56–10.96, respectively, Table 4).

**Table 4:** Kaplan-Meier survival analysis and Multivariate Cox regression analysis for OSCC patients

OSCC	Kaplan-Meier survival analysis unadjusted *p*-value	Multivariate Cox regression analysis adjusted *p*-value	Hazard’s ratio (H.R.)	95 % C.I.
Ep-ICD_Nuc_^+^	0.078	0.049	0.35	0.12–0.98
EpEx_Membrane_^+^	0.302	0.189	----	----
Ep-ICD_Nuc_ + EpEx_Membrane_^+^	**0.016**	**0.003**	**4.01**	**1.64–9.83**
Age	0.33	0.026	----	----
T classification	0.63	0.913	----	----
Nodal classification	**0.041**	0.633	----	----
Clinical stage	0.225	0.293	----	----
Extra capsular invasion	**<0.001**	**0.004**	**4.14**	**1.56–10.96**
Perineural involvement	0.828	0.102	----	----
Vascular involvement	0.355	0.552	----	----

## Discussion

Ever since the regulated intramembranous proteolysis of EpCAM was described as a novel mechanism of triggering oncogenic signaling by Maetzel et al. [34], investigation of Ep-ICD expression in human epithelial cancers for determination of its potential relevance to assist in the management of many human epithelial cancers has been undertaken. Our earlier preliminary study reported frequent Ep-ICD_Nuc_ and Ep-ICD_Cyt_ expression in ten different epithelial cancers, including a small number of head and neck cancers [36]. This first report did not examine the correlation of Ep-ICD_Nuc_ expression with clinical parameters or its prognostic utility in these cancers, nor did it evaluate the expression of these proteins in premalignant oral lesions with dysplasia prior to cancer development. The current study assessed the dynamic changes in Ep-ICD and EpEx expression in oral normal mucosa, dysplasia and OSCC to assess their relevance in oral tumorigenesis and potential suitability as marker in predicting clinical course and aggressiveness of head and neck cancer. Although expression of the full length EpCAM protein has been widely investigated in human malignancies, the expression and subcellular localization of its intracellular domain Ep-ICD has not been well characterized in clinical specimens. Our study demonstrated differences in expression of Ep-ICD and EpEx between normal, dysplastic and malignant oral tissues and their relationship with disease prognosis, providing valuable information as to their suitability as potential biological markers. Given the interest in the therapeutic potential of EpCAM targeted therapies in cancer management and the limited understanding of the role and expression pattern of Ep-ICD in oral cancer, our study helps to shed light on this widely-studied, yet not fully understood protein. Furthermore, our study is the first in-depth characterization of Ep-ICD expression in oral dysplasia and OSCC.

The increased expression of EpEx_Mem_ and Ep-ICD_Nuc_ in dysplasia in comparison with normal tissues suggests an overall upregulation of EpCAM expression as well as its increased proteolysis that would account for increased Ep-ICD_Nuc_. Interestingly, the increased regulated intramembranous proteolysis of EpCAM resulting in release of its cytoplasmic domain, Ep-ICD in colon carcinoma and its subsequent translocation to the nucleus has been demonstrated to trigger oncogenic signaling [34]. In our present study we observed increased Ep-ICD_Nuc_ in dysplasia and further increase in OSCC. Importantly, our findings on the follow up of patients with oral dysplasia demonstrate that patients with increased overall Ep-ICD (nuclear and cytoplasmic) developed cancer within a shorter time period as compared to those who did not show increased Ep-ICD; these observations are in accord with the proposed oncogenic function of Ep-ICD_Nuc_. Our findings are novel and of considerable clinical relevance in view of the fact that early prediction of malignant potential of oral epithelial dysplasia is crucial for precise clinical management of patients in early premalignant stages, prior to development of frank cancer.

In an earlier study, we reported that Ep-ICD_Nuc_ accumulation predicted poor prognosis in thyroid carcinomas and was elevated in patients with anaplastic tumors [36]. Notably, we observed that OSCC patients showing increased EpEx_Mem_ and Ep-ICD_Nuc_ had reduced disease free survival and poor prognosis as compared to patients who did not show this increase, suggesting that dynamic changes in EpEx_Mem_ and Ep-ICD_Nuc_ must be taken into account collectively to assess their prognostic utility in OSCC. It is important to note that our recent studies on prognostic relevance of Ep-ICD_Nuc_ and Ep-ICD_Cyt_ and EpEx in breast cancer and prostate cancer also demonstrated context dependent adaption of Ep-ICD in different human cancers [42, 43]. The recent report on EpCAM expression in early systemic esophageal cancer also supports our findings [16]. A dynamic expression of EpCAM was shown in esophageal cancer throughout tumor progression, where EpCAMhigh phenotypes correlated with proliferative stages, whereas EpCAMlow/negative phenotypes were associated with migration, invasion and dissemination, suggesting that differing expression levels of EpCAM occur during cancer progression and must be taken into consideration for therapeutic approaches and during clinical retrieval of disseminated tumor cells [29].

The discovery of the tumor-suppressive properties of EpCAM in some cancers has surprised many researchers, given its association with poor prognosis in many other cancers. Some studies have suggested the tumor microenvironment may be an important factor in dictating whether EpCAM will promote or inhibit tumor progression, particularly given its ability to mediate homophilic adhesive interactions between cells [5]. Furthermore, regulated intramembrane proteolysis of EpCAM and the associated oncogenic signalling by Ep-ICD may shed light on some of these observations as additional protein-protein interactions are uncovered [8, 44]. Recently, the endoplasmic reticulum aminopeptidase 2 (ERAP2), a proteolytic enzyme set in the endoplasmic reticulum (ER) has been shown to co-localize with EpCAM in the cytoplasm/ER where it plays a central role in the trimming of peptides for presentation by MHC class I molecules. This association between EpCAM and ERAP2 suggests a new mechanism of EpCAM processing and regulation of antigen presentation in breast cancer [45].

A major challenge is to predict the prognosis of OSCC patients effectively after completion of their primary treatment. In this context our study assumes importance, because of its retrospective nature, the large set of patients representing different stages of OSCC and long-term follow-up analysis. Our study uniquely based on sub-cellular compartment analysis of Ep-ICD and EpEx expression for correlation with clinical outcome, gave a more comprehensive insight into the clinical relevance of alterations in sub-cellular localization of a protein on disease outcome. Hence, our study emphasizes the importance of sub-cellular compartmental analysis of Ep-ICD and EpEx in membrane, cytoplasm and nucleus as compared to the overall protein expression reported in most of the earlier studies.

## Conclusions

In conclusion, we demonstrate overexpression of Ep-ICD occurs in early stages, in oral dysplasia and is sustained in cancer. Increased Ep-ICD in patients with oral dysplasia has the potential to serve as a biomarker to stratify patients at high risk of cancer development and enable early intervention in these patients for precise rigorous disease management prior to development of frank malignancy. Importantly, the combination of Ep-ICD_Nuc_ and EpEx_Mem_ can serve as a predictor of risk of recurrence in OSCC patients suggesting its potential to act as a prognostic marker to identify oral cancer patients who need more personalized post-treatment management.

## Abbreviations

95 % CI, 95 % confidence intervals; DFS, disease-free survival; EGF, epidermal growth factor; EpCAM, epithelial cell adhesion molecule; EpEX, extracellular domain of EpCAM; EpEX_Mem_, membrane EpEx; Ep-ICD, intracellular domain of EpCAM; Ep-ICD_Cyt_, cytoplasmic Ep-ICD; Ep-ICD_Nuc_, nuclear Ep-ICD; HR, hazard ratios; IHC, immunohistochemistry; MSH, Mount Sinai Hospital; NCCN, National Comprehensive Cancer Network; OSCC, oral squamous cell carcinoma; REMARK, Recommendations for Tumor Marker prognostic studies; TMA, tissue microarrays
